# Supplementary material for: Effect of chiropractic care on low back pain for active-duty military members: Mediation through biopsychosocial factors
Source: PLoS One. 2024 Oct 1;19(10):e0310642. doi: 10.1371/journal.pone.0310642 (PMC11444394; doi:10.1371/journal.pone.0310642)
Supplement: S1 Table — (PDF) [file pone.0310642.s001.pdf]

Variable name (modeled)	Scale	Recall Period	Question(s)	Response Options
**Pain Interference (outcome)**	Likert (1-5)	7 days	“How much did pain interfere with your day-to-day activities?”, “How much did pain interfere with work around the home?”, “How much did pain interfere with your ability to participate in social activities?”, “How much did pain interfere with your household chores?”	“Not at all”, “A little bit”, “Somewhat”, “Quite a bit”, “Very much”
**Pain Intensity (outcome)**	0-10	7 days	Rate your pain on average	0 (no pain) to 10 (worst imaginable pain)
**Sex (baseline covariate)**	Binary	None	Sex:	Male, Female
**Low back pain duration (baseline covariate)**	Ordinal Categorical	Varies	How long ago did your current episode of low back pain begin?	“Less than 7 days ago”, “7 days to less than 16 days ago”, “16 days to less than 1 month ago”, “1-3 months ago”, “More than 3 months and less than 6 months ago”, “6 months to less than 1 year ago”, “1 year or more ago”
