# Supplementary material for: Effect of chiropractic care on low back pain for active-duty military members: Mediation through biopsychosocial factors
Source: PLoS One. 2024 Oct 1;19(10):e0310642. doi: 10.1371/journal.pone.0310642 (PMC11444394; doi:10.1371/journal.pone.0310642)
Supplement: S2 Table — UMC: usual medical care; UMC+CC: usual medical care plus chiropractic care. (PDF) [file pone.0310642.s002.pdf]

Variable name (modeled)	Time (week)	UMC (n=375) n (%)	UMC+CC (n=375) n (%)	Total (n=750) n (%)
**Pain Interference (outcome)**	12	288 (76.8)	286 (76.3)	574 (76.5)
**Pain Intensity (outcome)**	12	288 (76.8)	286 (76.3)	574 (76.5)
**Physical Function (mediator)**	6	316 (84.3)	323 (86.1)	639 (85.2)
**Sleep Disturbance (mediator)**	6	316 (84.3)	323 (86.1)	639 (85.2)
**Fatigue (mediator)**	6	316 (84.3)	323 (86.1)	639 (85.2)
**Anxiety (mediator)**	6	315 (84.0)	323 (86.1)	638 (85.1)
**Depression (mediator)**	6	316 (84.3)	323 (86.1)	639 (85.2)
**Social Roles (mediator)**	6	316 (84.3)	321 (85.6)	637 (84.9)
